# Impact of next-generation vehicles on tropospheric ozone estimated by chemical transport model in the Kanto region of Japan

**DOI:** 10.1038/s41598-019-40012-y

**Published:** 2019-03-05

**Authors:** Hiroo Hata, Kenichi Tonokura

**Affiliations:** 1 Tokyo Metropolitan Research Institute for Environmental Protection 1-7-5, Sinsuna, Koto-ku, Tokyo 136-0075 Japan; 2 Graduate School of Frontier Sciences, The University of Tokyo, 5-1-5 Kashiwanoha, Kashiwa, Chiba, 277-8563 Japan

## Abstract

The plans to introduce next-generation hybrid and zero-emission vehicles in the market are now enacted by governments in many countries to manage both global warming and air pollution problems. There are only a few studies evaluating the effects of the next-generation vehicles on the changes in concentrations of ozone generated by the photochemical reactions between volatile organic compounds and nitrogen oxides (NO_x_). To evaluate these changes, we performed chemical transport modeling in the Kanto region, Japan in the summer of 2013. The results show that if the vehicles are substituted by hybrid vehicles, average ozone concentrations increase in urban areas and decrease in suburban areas due to NO_x_ titration. Substitution with zero-emission passenger vehicles decreases the concentrations in both urban and suburban areas. Substitution with both hybrid and zero-emission passenger and heavy-duty vehicles highly increases the concentrations in urban areas. Using the model results, we also discuss the effect of ozone concentration changes on premature mortality of humans in summer. The results suggest that, in some cases the introduction of next-generation vehicles might exasperate ozone concentrations, even leading to 5 to 10 times higher premature mortality during the summer compared to that of influenza and heat stroke in Japan.

## Introduction

The large concentrations of ozone in the atmosphere are still a significant problem in many countries because of the negative effects of ozone on human health and crops; thus, several environmental measures to control the generation of ozone have been proposed by governments. The Japanese government proposed an ozone concentration limit of 0.06 ppm averaged over one hour^1^. Ozone is generated by photochemical reactions between nitrogen oxides (NO_x_) and volatile organic compounds (VOCs)^2^; thus, it is rational to decrease the NO_x_ and VOC emissions to reduce ozone concentrations in the atmosphere, and several studies theoretically evaluated ozone concentration with the implementation of decreasing anthropogenic NO_x_ and VOC emissions using the chemical transport model. Moghani *et al*.^3^ evaluated the contribution of anthropogenic sources of NO_x_ and VOCs to tropospheric ozone in six states in the United States. The results suggested that the implementation of anthropogenic emission sources is effective at reducing ozone concentrations in windy environments. This means that the emission sources of the advection of ozone should be targeted to reduce anthropogenic sources. Zawacki *et al*.^4^ evaluated the mobile source contributions to ozone and PM_2.5_ concentrations in near future, specifically 2025. The study suggested that heavy-duty, light-duty, and off-road diesel vehicles contribute considerably to ozone and fine particulate matter (PM_2.5_) generation in urban areas in the United States.

Vehicles are one of the major sources of NO_x_ and VOCs and new technologies to mitigate pollutant emissions have been applied to vehicles. For example, three-way catalysts, which convert carbon monoxide (CO), NO_x_, and VOCs from tailpipe emissions to harmless gases, are attached to gasoline vehicles^5^, and selective catalytic reduction systems (SCRs) or NO_x_ storage reduction catalysts, which can reduce NO_x_ from tailpipe emissions, are attached to diesel vehicles^6^. Technologies to trap VOCs in evaporative emissions from gasoline vehicles or the refueling process are also applied in many countries^7–9^. Despite the application of these technologies, the problem of NO_x_ and VOCs emissions from vehicles still remains and it is important to understand the effect of vehicle emissions on ozone and PM_2.5_pollution in the atmosphere.

The introduction of next-generation vehicles, which are generally composed of hybrid and zero-emission vehicles (electric and fuel-cell vehicles), to the vehicle markets has been strongly promoted in many countries to mitigate the effects of global warming caused by CO_2_ emissions and to control air pollution. The French government announced that France will end sales of gasoline and diesel vehicles by 2040^10^. The United Kingdom, China, and other countries also announced plans to phase out gasoline and diesel vehicles in the middle of the 21^st^ century^11,12^. The Japanese government is planning to introduce next-generation vehicles in the market but a detailed introduction scenario is still under consideration. The introduction of next-generation vehicles is expected to decrease NO_x_ and VOC emissions, which will affect ozone concentrations in the atmosphere. Jacobson^13^ evaluated ozone concentration changes using a chemical transport model after modern diesel passenger vehicles were introduced in the United States. The results of Jacobson’s research suggested that the introduction of modern diesel vehicles to the United States contributed to a decrease in ozone concentrations due to atmospheric conditions in urban areas in the United States. Previous studies analyzed the effects of substituting gasoline passenger vehicles only by diesel passenger vehicles, but similar calculations are needed for the next-generation vehicles replacing both passenger and heavy-duty vehicles, owing to the global demand for next-generation vehicles. In Japan, more than 90% of the passenger cars are gasoline-powered and almost all heavy-duty vehicles are diesel-powered; therefore, it is important to evaluate the effect of substituting so many vehicles by next-generation vehicles on tropospheric ozone concentrations.

In this study, we evaluated the changes in ozone concentrations caused by the introduction of next-generation vehicles (hybrid and zero-emission vehicles) in the Kanto region that includes the Tokyo metropolitan area in Japan using a chemical transport model. The results were also used to analyze the effect of these changes on human premature mortality. To our knowledge, this study is the first to evaluate the effect on ozone concentrations from introducing next-generation vehicles. The results of this study will help to predict the environmental impact from introducing next-generation vehicles on a global scale.

## Results

### Calculation results for each scenario

**Figure 1:**
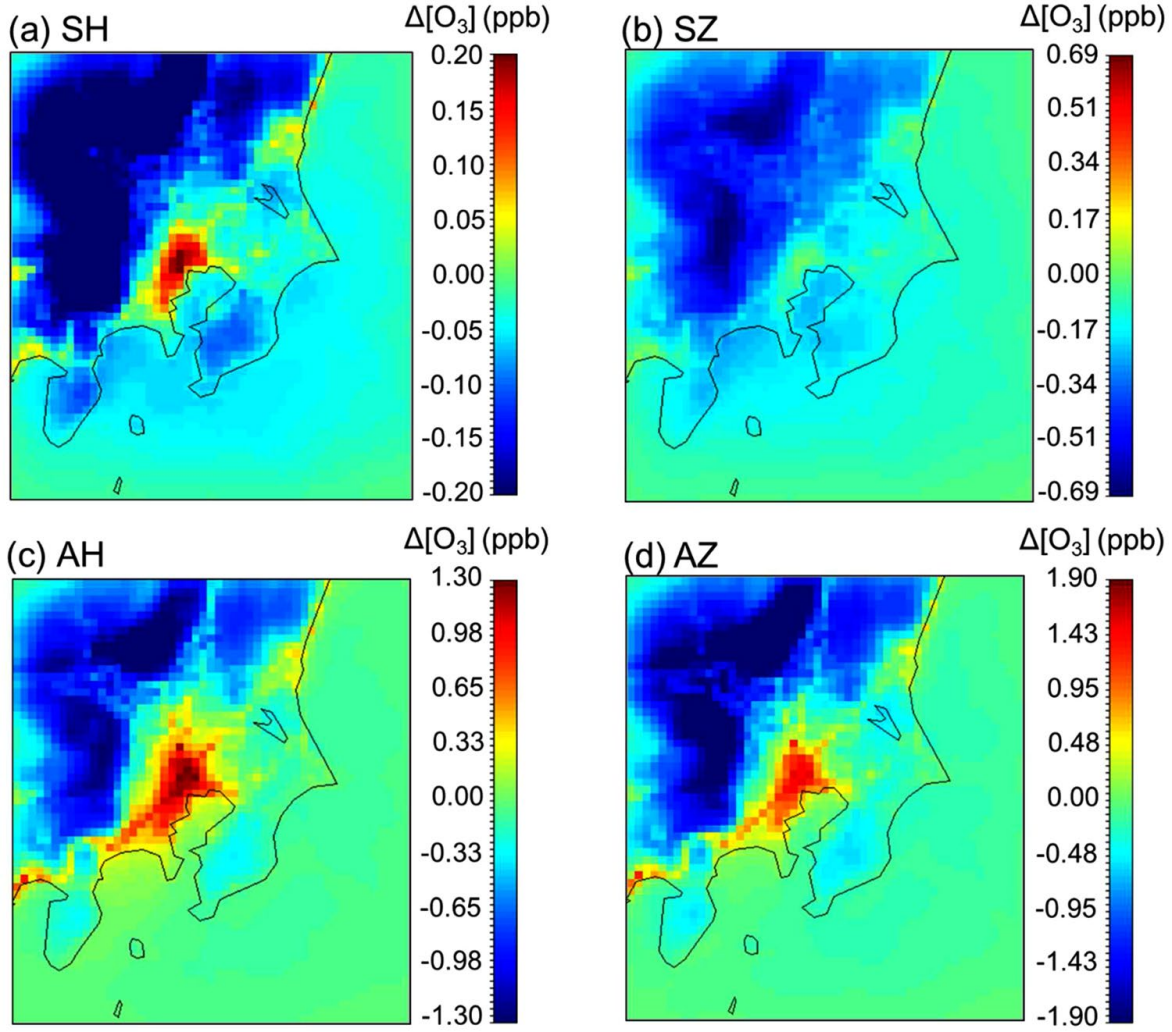
The average changes in ozone concentrations during the study period after replacing vehicles by next-generation vehicles in the Kanto region of Japan.

Figure 1(a,b) show the changes in ozone concentrations if all gasoline fuel passenger vehicles are replaced by gasoline hybrid and zero-emission vehicles in the Kanto region. The model period is two months from July 1 to August 31, 2013 and all the results are the average values over this period. The results of scenario SH show that if all the gasoline passenger vehicles are replaced by gasoline hybrid vehicles, the concentrations of ozone in the center and the surrounding area of Shinjuku will increase by less than 0.2 ppb although the emissions of NO_x_, CO, and VOCs will decrease. In contrast, scenario SZ shows that ozone concentrations will decrease with the introduction of zero-emission vehicles to replace gasoline passenger vehicles. Figure 1(c,d) show the results of the ozone concentration changes when all passenger and diesel heavy-duty vehicles are replaced by hybrid and zero-emission vehicles. The analysis term is the same as that in Figure 1(a,b). If all passenger and heavy-duty vehicles are replaced by hybrid or zero-emission vehicles, ozone concentrations will increase in Shinjuku and Yokohama, which are the metropolitan areas, and decrease in Utsunomiya and Maebashi, which are the suburban areas in the model domain.

Figure S9 in Supplementary Information (SI) shows the average ozone concentrations of the base scenario (BASE). The value of ozone concentrations ranges from 26 to 56 ppb in the Kanto area.

### Model validation

The validity of the results of CMAQ calculations compared to the observed data is discussed in Text S10 in SI.

## Discussion

### Effect of introducing next-generation PASSENGER vehicles on ozone

**Figure 2:**
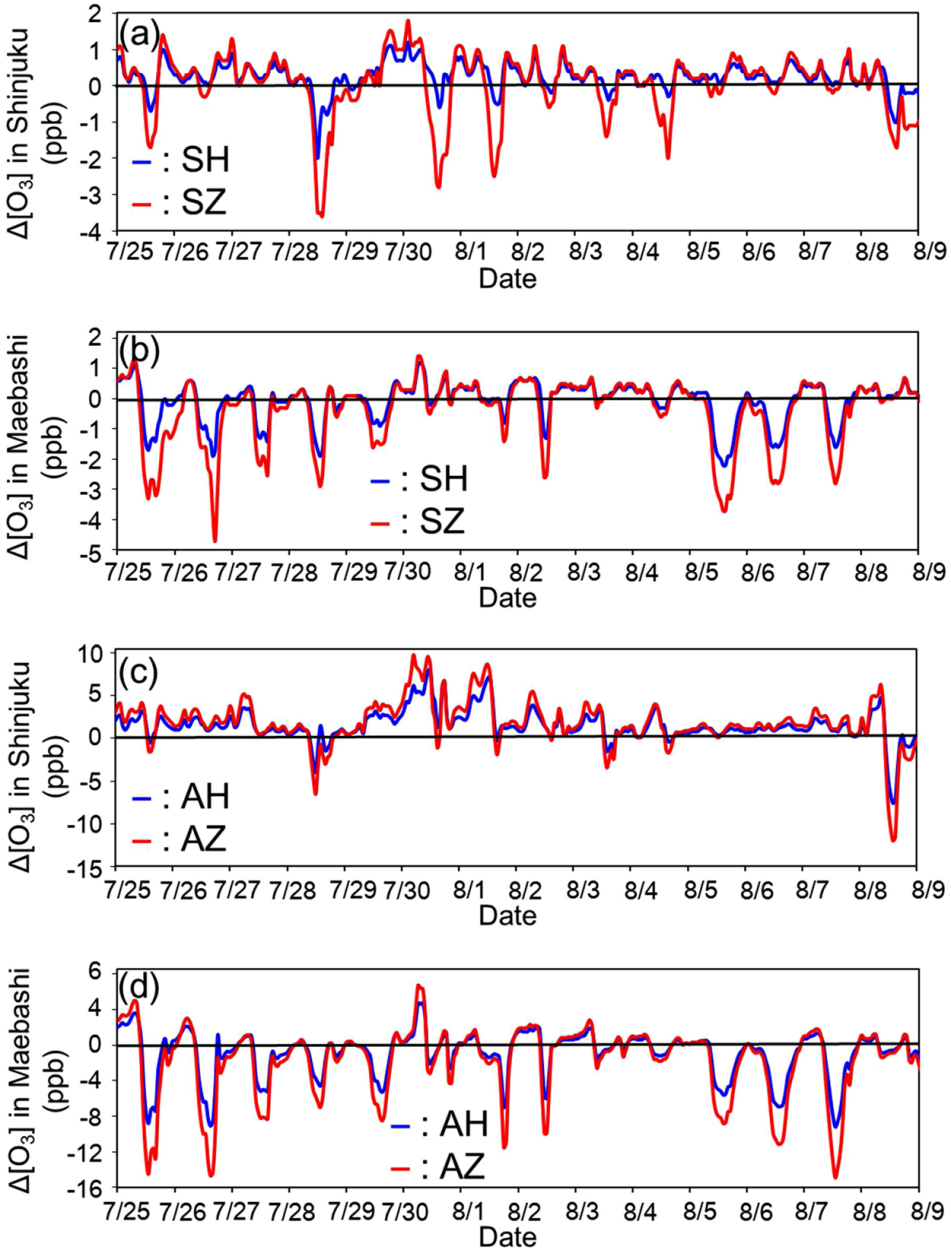
Temporal changes in ozone concentrations from the base scenario (BASE) to the next-generation vehicle introduction scenarios (**a**) SH and SZ in Shinjuku, (**b**) SH and SZ on Maebashi (**c**) AH and AZ in Shinjuku and (**d**) AH and AZ in Maebashi.

Figure 1(a,b) show that although the introduction of both hybrid and zero-emission vehicles reduces the emissions of NO_x_ and VOCs, the changes in ozone concentrations in Shinjuku are different between scenario SH (passenger vehicles are substituted by hybrid vehicles) and SZ (passenger vehicles are substituted by zero-emission vehicles). To discuss the reasons behind this difference, the time profile of ozone concentration changes from July 25 to August 9 in Shinjuku for scenarios SH and SZ is displayed in Figure 2(a). Ozone concentrations in Shinjuku decrease during the day and increase at night in both SH and SZ scenarios. Previous studies of photochemical models and field measurements showed that urban areas are VOC-sensitive. Specifically, the photochemical generation of ozone is proportional to VOC concentrations and inversely proportional to NO_x_ concentrations^14,15^. By contrast, rural areas are NO_x_-sensitive. That is, ozone concentrations strongly depend on NO_x_ concentrations and are less dependent on VOCs. In this study, Shinjuku (Tokyo) and Yokohama (Kanagawa) are the metropolitan areas, and Chiba (Chiba) and Saitama (Saitama) are the urban areas. The remaining three prefectures are suburban areas. Because Shinjuku is VOC-sensitive, VOC emission reductions by the introduction of hybrid and zero-emission vehicles decrease ozone generation during the day. However, atmospheric ozone at night, which is generated in the daytime or comes from other places by advection, is consumed by NO included in NO_x_ by the following chemical reaction (NO_x_ titration).

$${{\rm{O}}}_{3}+{\rm{NO}}\to {{\rm{O}}}_{2}+{{\rm{NO}}}_{2}$$

Ozone concentrations increase in the nighttime in scenarios SH and SZ because NO_x_ emissions from vehicles decrease with the introduction of hybrid and zero emission vehicles and the effect of NO_x_ titration is weakened at night. In the hybrid vehicle introduction scenario, SH, the increase in nighttime ozone concentrations is higher than the decrease in daytime ozone concentrations because of the insufficient reduction of VOC emissions in VOC-sensitive regimes. While the average ozone concentrations increase in Shinjuku, the introduction of next-generation vehicles is considered effective in meeting the environmental standards. Recent environmental standards are defined as the maximum 8-h average concentration in the United States and European Union and maximum 1-h average concentration in Japan. The maximum ozone concentration occurs in the daytime and the introduction of next-generation vehicles may decrease the daytime ozone concentrations, helping the governments to meet the environmental standards. Furthermore, people are more active outside in the daytime and thus the exposure to tropospheric ozone is larger during the day than that at night. The introduction of next-generation passenger vehicles, therefore, is expected to have positive human health consequences in metropolitan areas. Figures S11–S13 in SI show the time profiles of ozone concentrations for scenarios SH and SZ in Yokohama, Chiba, and Saitama, respectively. These cities are metropolitan and urban areas and the trends are similar to the results of Shinjuku.

In Figure 1(a,b), ozone concentrations decrease in the suburban areas of the Kanto region, Utsunomiya and Maebashi. The suburban areas are NO_x_-sensitive regimes; thus, ozone concentrations are directly affected by the decrease in NO_x_ concentrations following the introduction of hybrid and zero-emission vehicles. Figure 2(b) shows the time profile of ozone concentration changes in Maebashi for both SH and SZ scenarios. The increase in ozone concentrations at night, caused by NO_x_ titration, is also confirmed for scenarios SH and SZ in Maebashi but the values are smaller than the decrease in ozone concentrations in the daytime. Therefore, the introduction of next generation passenger vehicles in suburban areas in Kanto is expected to be highly effective in reducing ozone concentrations.

The increase in ozone concentrations in Mito is evident in Figure 1(a,b) although Mito is also a suburban area such as Utsunomiya and Maebashi. Figure S16 shows the emission inventories of NO_x_ from industries, vehicles, and all emission sources. The industrial area in the northern part of Mito is associated with large NO_x_ emissions from factories and vehicles. Therefore, atmospheric conditions in Mito are similar to those in metropolitan or urban areas (VOC-sensitive), increasing the ozone concentrations. Figure S15(a) in SI shows the trends in ozone concentrations in Mito for scenarios SH and SZ. The introduction of next-generation passenger vehicles is not quite effective in controlling ozone concentrations in the region.

### The effect of introducing both next generation PASSENGER and HEAVY-DUTY vehicles on ozone

Heavy-duty vehicles are one of the most important sources of NO_x_ emissions and approximately 20% of anthropogenic NO_x_ emissions in 2011 come from heavy-duty diesel vehicles in Japan^16^. Thus, the decrease in tailpipe emissions from heavy-duty vehicles strongly affects the ozone concentrations in both VOC- and NO_x_-sensitive regimes. Figure 1(c,d) show that ozone concentrations increase dramatically in the center of the Tokyo metropolitan area (Shinjuku) if all the vehicles are substituted by hybrid and zero-emission vehicles and they decrease in the suburban areas of Kanto. Figure 2(c) shows the time profile of ozone concentration changes from the base scenario to the hybrid and zero-emission vehicle introduction scenarios in Shinjuku. Ozone concentrations increase most of the time for scenarios AH and AZ (Fig. 2(c)). If heavy-duty diesel vehicles are replaced with hybrid and zero-emission vehicles, a significant reduction in NO_x_ emissions will be observed. Tokyo is a VOC-sensitive regime; thus, abrupt NO_x_ concentration decreases will increase the ozone concentrations during the day. Furthermore, because of the decrease in the NO_x_ titration effect, ozone concentrations will also increase at night. These factors will increase the average ozone concentrations in Shinjuku for scenarios AH and AZ, as well as in Yokohama, Chiba, Saitama and Mito, which have similar atmospheric conditions to that of the Tokyo metropolitan area. The time profiles of ozone concentrations for these regions are illustrated in Figures S11, S12, S13 and S15 in SI.

In scenarios SH and SZ, ozone concentrations in the suburban areas of Utsunomiya and Maebashi decrease because of the NO_x_-sensitive conditions. The time profile of ozone concentrations in Maebashi is displayed in Figure 2(d). In scenarios AH and AZ, ozone concentrations in Maebashi increase because of the NO_x_ titration at night but daytime ozone levels decrease significantly as well as the average concentration of ozone in suburban areas. These results suggest that the introduction of next-generation passenger and heavy-duty vehicles is highly effective in suburban areas of Utsunomiya and Maebashi in reducing ozone concentrations. The time profile of ozone concentrations in Utsunomiya for scenarios AH and AZ is illustrated in Figure S14 in SI.

### Effects of changes in ozone concentrations on human premature mortality in summer

In the previous subsection, the ozone concentration changes induced by the introduction of next generation vehicles were described. To evaluate the effect of ozone concentration changes on human health in summer months, premature mortality rate was calculated for each scenario in seven cities in Japan. The change in mortality (% change in total number of ozone-exposure-related deaths), Δ*P*, is proposed as follows^17^:

1 $${\rm{\Delta }}P={\rm{sgn}}({\rm{\Delta }}[{{\rm{O}}}_{3}])\cdot (1-{e}^{-\beta \cdot |{\rm{\Delta }}[{{\rm{O}}}_{3}]|})\times 100$$

where Δ[O_3_] is the change in ozone concentration and *β* is the parameter estimated by epidemiological studies. The function sgn is the sign function. *β* is calculated using the following equation.

2 $$\beta =\frac{\mathrm{ln}(RR)}{{\rm{\Delta }}{[{{\rm{O}}}_{3}]}_{RR}}$$

where *RR* is the relative risk of death from ozone exposure and Δ[O_3_]_*RR*_ is the daily average ozone concentration change. The *RR* value was obtained from a previous study of Chen *et al*.^18^ and the value in Japan in summer was determined to be 0.27% increase in total mortality rate with Δ[O_3_]_*RR*_ = 10 μg/m^3^ for 8-h maximum ozone concentration, which corresponds to 0.51% increase in total mortality rate in daily average ozone.

Table 1 shows the calculated value of premature mortality change (%) for each scenario in seven capital cities in the Kanto region. The plus sign means the increase and minus sign means the decrease in premature mortality. As described in the previous subsection, when the hybrid and zero-emission vehicles are introduced in the Kanto region, the ozone concentrations are estimated to increase in metropolitan and urban area and decrease in suburban area (except for scenario SH for Mito), causing an increase in premature mortality in metropolitan area and a decrease in premature mortality in suburban area. Introducing hybrid and zero-emission vehicles for both passenger and heavy-duty vehicles is estimated to increase premature mortality in metropolitan areas including Shinjuku and Yokohama. Meanwhile, the annual rates of mortality from heat stroke and influenza in Japan were approximately 0.05% and 0.015%, respectively^19,20^. Some of the absolute values of ozone-exposure-related premature mortality described in Table 1 are 5 to 10 times higher than heat stroke and influenza-related mortality. This means that the introduction of hybrid and zero-emission vehicles in the Kanto region will have significant impacts on human health. Therefore, careful consideration of the effects of replacing the current fleet by next-generation vehicles on air quality and human health is required.

**Table 1:** The changes in premature mortality (%) related to ozone concentration changes caused by the introduction of next-generation vehicles.

Scenario	Shinjuku	Yokohama	Chiba	Saitama	Utsunomiya	Maebashi	Mito
SH	0.03	0.01	0.02	0.02	0.01	−0.01	0.01
SZ	0.01	−0.01	0.01	−0.01	−0.02	−0.04	0.00
AH	0.14	0.07	0.07	0.09	−0.04	−0.09	0.02
AZ	0.16	0.07	0.07	0.07	−0.11	−0.18	0.01

### Overall summary of discussion

The results indicate that the introduction of next-generation vehicles is sometimes less effective at controlling ozone, especially in metropolitan areas. Indeed, this study focused exclusively on the change in emissions as a result of next-generation vehicles. However, it is well known that the prevalence of zero-emission vehicles will incur higher demands from thermal power plants. Importantly, this study did not consider energy strategies that might be undertaken by power plants to deal with this increased demand. Nevertheless, power plants in Japan are mainly in coastal areas, so the changes in ozone concentrations evaluated in this study are not expected to differ dramatically, regardless of such strategies. In future research, we shall include the effect of emissions from power plants in order to better assess the effects of next-generation vehicles.

## Methods

### Chemical transport model

**Figure 3:**
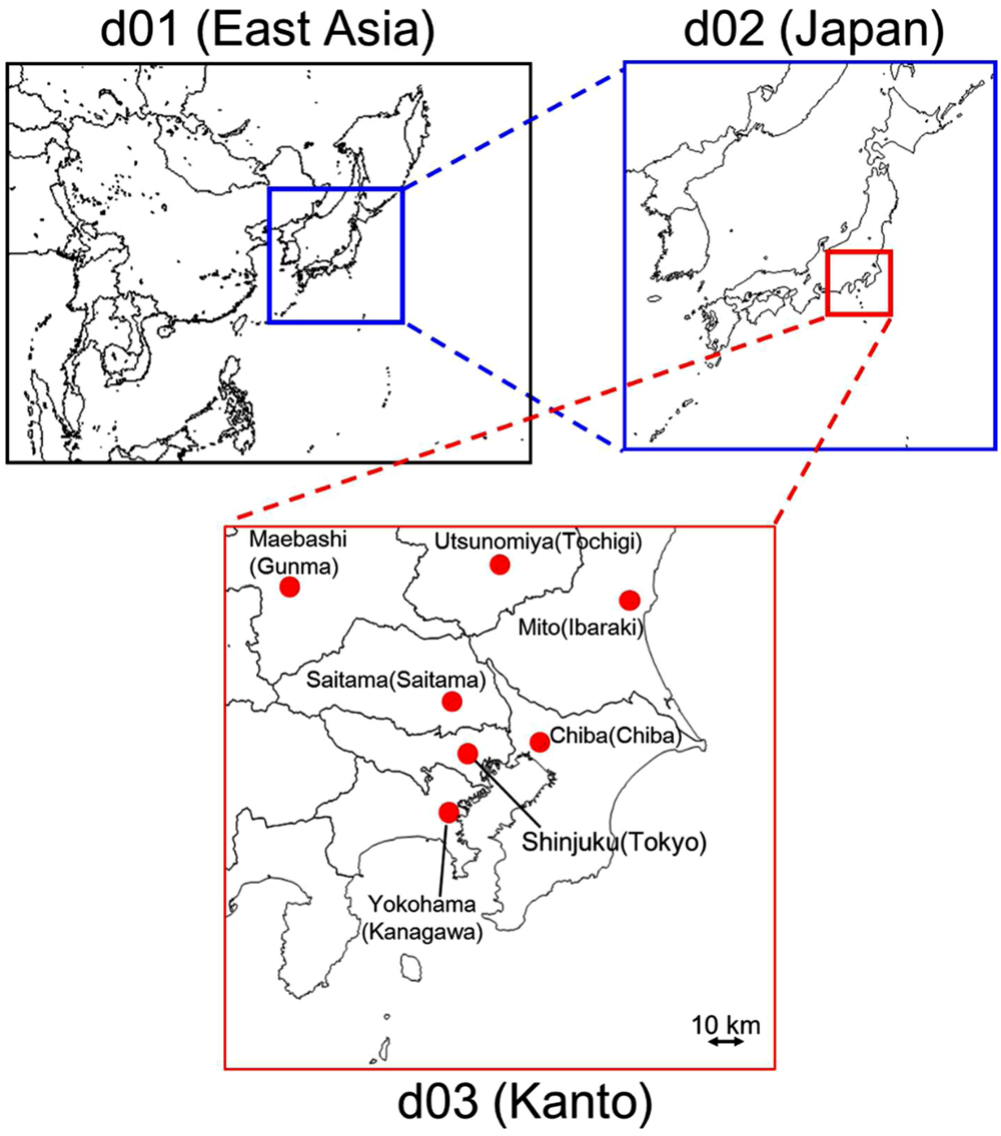
Model domain. The locations of the capital cities used in the analysis are mapped in d03 (prefecture name is written in parenthesis).

The Weather Research and Forecasting model (WRFv3.7.1)^21^ was used for meteorological evaluation used in the chemical transport model and the modeling period was December 1, 2012–April 31, 2014. The details of the calculation conditions of WRF in this study are listed in Table S8**-**1 in SI. The community Multiscale Air Quality Modeling System (CMAQv5.2)^22^ was used to calculate the concentrations of anthropogenic and non-anthropogenic pollutants in the atmosphere. The details of the calculation conditions of CMAQ are listed in Table S8**-**2 in SI. Figure 3 shows the model domain^23^ and the three nested domains, d01 (East and Southeast Asia with a mesh size of 45 × 45 km), d02 (all Japan with a mesh size of 15 × 15 km), and d03 (Tokyo metropolitan area with a mesh size of 5 × 5 km). There are seven prefectures inside d03, Tokyo, Kanagawa, Chiba, Saitama, Tochigi, Gunma, and Ibaraki. Tokyo is situated on the largest plain in Japan, the Kanto Plain. The population in the Kanto region is about one third (43 million) of Japan and is concentrated in Tokyo. Several expressways including the metropolitan expressway pass through the model domain. Major industrial areas are situated in the suburbs of Tokyo including the Keihin and the Keiyo industrial areas located on the coast of Tokyo Bay. There are mountainous areas over the northern and western parts of the Kanto plain.

Emission inventories for outside Japan (d01) were determined using HTAPv2.2^24^ (anthropogenic), GFEDv4.1^25^ (biomass burning), MEGANv2.1^26^ (biogenic VOCs), and AeroCom^27^ (volcanoes). Emission inventories within Japan (d02 and d03) were determined using the JATOP emission inventory-vehicle emission estimation model (JEI-VEM)^28^ (vehicles and other anthropogenic), Sasakawa Peace Foundation (SPF)^29^ (Ship), MEGANv2.1^26^ (biogenic VOCs), and Japan Meteorological Agency (JMA)^30^ (volcanoes). SAPRC07^31^ was used as the principle mechanism to calculate the chemical rate coefficients for gas-phase chemistry and AERO6 module in CMAQ for aerosol chemistry. The calculation results of MOZART-4/GEOS-5^32^ was used for the boundary condition of d01. The modeling period was the summer season from June 26 to August 31, 2013. The period from June 26 to 30 was treated as pre-conditioning term of the calculation and the analyzed period was from July 1 to August 31. We chose the summer season because the ozone concentrations are higher than the environmental standards of Japan especially in the summer because of the intense sunlight and high temperatures.

To evaluate the effect of introducing next-generation vehicles on air quality in Japan, we assumed five scenarios, BASE, SH, SZ, AH, and AZ and the details of each scenario are described in Table 2. SH and SZ are the scenarios for substituting passenger vehicles by next-generation vehicles. AH and AZ are the scenarios changing both passenger and heavy-duty vehicles to next-generation vehicles. BASE is the base scenario, which represents the air quality in Japan in 2013. The scenarios were built by multiplying the corresponding emission factors, calculated by the results of chassis dynamometer measurements, by the values of the base scenario.

**Table 2:** The details of next generation vehicle introduction ratio for each calculation scenario.

Scenario	Passenger vehicles			Heavy-duty vehicles		
	Fuel	Hybrid	Zero-emission	Fuel	Hybrid	Zero-emission
BASE	0.83	0.17	0	1	0	0
SH	0	1	0	1	0	0
SZ	0	0	1	1	0	0
AH	0	1	0	0	1	0
AZ	0	0	1	0	0	1

### Emission factors and VOCs compositions after next-generation vehicles are introduced

The vehicle exhaust emissions are composed of two factors: tailpipe emissions and evaporative emissions. Tailpipe emissions contain the exhaust gas generated by engine combustion when vehicle is running or idling. Usually, the exhaust gas from engine combustion is detoxified by the catalyst attached to the vehicle exhaust pipe but not all the pollutants can be completely removed. When the vehicle is started after sitting for a long time (more than 6 h) and the temperature of the catalyst is low, the exhaust gas from engine combustion is not purified and large quantities of pollutants are emitted to the atmosphere (cold-start emissions). Tailpipe emissions other than cold-start emissions are called hot-start emissions. Evaporative emissions include running loss, hot soak loss, diurnal breathing loss, and refueling loss. Running loss is evaporative emissions from warmed fuel-related parts when the vehicle is running. Hot soak loss is evaporative emissions from warmed fuel-related parts when the vehicle stops running and are soaked in the atmosphere within an hour. Diurnal breathing loss is evaporative emissions from the empty space of a fuel tank with the expansion of vapors caused by diurnal temperature changes, which occurs during long-time parking. Refueling loss is evaporative emissions when the refueling is conducted and vapors inside the empty space of the fuel tank are pushed out to the atmosphere. Evaporative emissions are released only from gasoline vehicles because of the high volatility of gasoline; evaporative emissions from diesel vehicles are almost zero. Based on this background information, emission factors to build the next-generation vehicles were constructed using the following procedure.

#### Tailpipe emissions

The pollutants used in this study are NO_x_, CO, and VOCs because these pollutants directly affect the photochemical generation of ozone. Emission factors for these three pollutants were determined by the experimental results of this study for gasoline passenger vehicles and hybrid passenger vehicles. If the value of NO_x_ emissions from gasoline passenger vehicles is *α* and that from hybrid vehicles is *β*, the emission factor of introducing hybrid vehicles for NO_x_ is calculated as *β*/*α*. The emission factor is set to zero for zero-emission vehicles. The calculated emission factors for both hot-start and cold-start emissions for hybrid vehicles are shown in Figures S3-1 and S3-2 in SI. We assume that the emission factors of heavy-duty hybrid vehicle are the same as those of hybrid passenger vehicles because experiments for hybrid heavy-duty vehicles were not conducted in this study. The NO/NO_x_ ratio of the vehicle exhaust was set to 0.95 based on a previous study^33^. The composition of VOCs from tailpipe emissions, required by the chemical kinetics mechanisms of SAPRC07, was determined by the experimental results of this study from GC-MS/FID and LC-MS and the distribution of the composition in SAPRC07 is described in Tables S7-1 and S7-2.

#### Evaporative emissions

The amount of evaporative emissions from running loss, hot soak loss, and diurnal breathing loss is assumed to be the same between gasoline vehicles and gasoline hybrid passenger vehicles because both vehicles include gasoline in the fuel tank. The amount of refueling loss-related VOC emissions depends on the frequency of refueling and the frequency is proportional to fuel consumption (L/km). Thus, if fuel consumption from gasoline passenger vehicles is *γ* and that from hybrid vehicles is *δ*, the emission factor of refueling emissions for introducing hybrid vehicles is *δ*/*γ*. The emission factor is set to zero for zero-emission vehicles. The composition of VOCs from evaporative emissions for SAPRC07 was obtained from our previous study^6^ and the details are listed in Table S7-3 of SI.

### Chassis dynamometer measurements

We obtained the emission factors for the hybrid vehicle introduction scenario and the composition of tailpipe emissions from chassis dynamometer experiments. All the measurement-related data and information are presented in SI.

## Supplementary information

Impact of next-generation vehicles on tropospheric ozone estimated by chemical transport model in the Kanto region of Japan

## Data Availability

The modeling data and chassis dynamometer experiment data are available from the authors upon request.
