# Author Correction: Impact of next-generation vehicles on tropospheric ozone estimated by chemical transport model in the Kanto region of Japan

**DOI:** 10.1038/s41598-020-59300-z

**Published:** 2020-02-06

**Authors:** Hiroo Hata, Kenichi Tonokura

**Affiliations:** 1 Tokyo Metropolitan Research Institute for Environmental Protection 1-7-5, Sinsuna, Koto-ku, Tokyo 136-0075 Japan; 2 Graduate School of Frontier Sciences, The University of Tokyo, 5-1-5 Kashiwanoha, Kashiwa, Chiba 277-8563 Japan

Correction to: *Scientific Reports* 10.1038/s41598-019-40012-y, published online 05 March 2019

The Supplementary Figure file that accompanies this Article contains an error in Supplementary Figure S1-1, whereby it was incorrectly extended to 1300s. The correct Figure S1-1 appears below as Figure 1.

**Figure 1:**
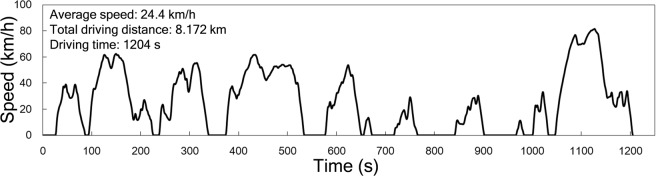
.

Additionally, the Supplementary Information file that accompanies this Article contains errors in Supplementary Table S8-2.

The row entitled “Initial concentration (d02)” under the column “CMAQv5.2” should read:

“Nested from d01”

Furthermore, the row entitled “Initial concentration (d03)” under the column “CMAQv5.2” should read:

“Nested from d02”

