# Supplementary material for: Impact of next-generation vehicles on tropospheric ozone estimated by chemical transport model in the Kanto region of Japan
Source: Sci Rep. 2019 Mar 5;9:3573. doi: 10.1038/s41598-019-40012-y (PMC6400957; doi:10.1038/s41598-019-40012-y)
Supplement: Supplementary file 1 — Impact of next-generation vehicles on tropospheric ozone estimated by chemical transport model in the Kanto region of Japan [file 41598_2019_40012_MOESM1_ESM.docx]

*Supplementary Information*

**Impact of next-generation vehicles on tropospheric ozone estimated by chemical transport model in the Kanto region of Japan**

*Hiroo Hata^a, b, *^ and Kenichi Tonokura^b,*^*

*^a^ Tokyo Metropolitan Research Institute for Environmental Protection 1-7-5, Sinsuna, Koto-ku, Tokyo 136-0075, Japan*

*^b^ Graduate School of Frontier Sciences, The University of Tokyo, 5-1-5 Kashiwanoha, Chiba 277-8563, Japan*

^*^Corresponding author. E-mail: hata-h@tokyokankyo.jp; Tel: +81-3-3699-1369

E-mail: tonokura@k.u-tokyo.ac.jp; Tel: +81-4-7136-4706

**CONTENTS**

| Text S1 | Measurement of exhaust emissions using chassis dynamometer |
| --- | --- |
| Figure S1-1 | Time profile of JC08 |
| Figure S1-2 | Time profiles of Tokyo metropolitan area driving patterns No.1-6 |
| Figure S1-3 | Time profiles of Tokyo metropolitan area driving patterns No.7-12 |
| Text S2 | Composition analysis of VOCs from tailpipe exhaust emissions |
| Table S2-1 | Experimental setup for GC-FID |
| Table S2-2 | Experimental setup for GC-MS |
| Table S2-3 | Experimental setup for LC-MS |
| Text S3 | Tailpipe emissions from gasoline and hybrid passenger vehicles |
| Figure S3-1 | Relationships between hot-start tailpipe emissions (or fuel consumption) and vehicle velocity |
| Figure S3-2 | Comparison of cold-start emissions of JC08 between gasoline and hybrid vehicles |
| Text S4 | Determination of tailpipe emission factors for gasoline and hybrid passenger vehicles |
| Table S4-1 | Emission factors of the hybrid vehicle introduction scenario |
| Table S4-2 | Emission factors of zero-emission vehicle introduction scenario |
| Figure S4 | The average distribution of vehicle velocity in Tokyo area |
| Text S5 | VOC composition from tailpipe emission |
| Table S5-1 | The average VOC composition of tailpipe emissions from gasoline and hybrid passenger vehicles |
| Table S5-2 | The average VOC composition of tailpipe emissions from diesel heavy-duty vehicles |
| Text S6 | VOC composition of evaporative emissions |
| Table S6 | The average VOC composition ratio from evaporative emissions of HSL and DBL |
| Text S7 | Distribution of VOC composition analysis results in SAPRC07 form |
| Table S7-1 | VOC distribution of tailpipe emissions from passenger vehicles in SAPRC07 form |
| Table S7-2 | VOC distribution of tailpipe emissions from heavy-duty vehicles in SAPRC07 form |
| Table S7-3 | VOC distribution of evaporative emissions in SAPRC07 form |
| Text S8 | Calculated conditions of WRF and CMAQ |
| Table S8-1 | The calculation conditions of WRF |
| Table S8-2 | The calculation conditions of CMAQ |
| Text S9 | Calculation results of base scenario |
| Figure S9 | Calculation results of base scenario for average ozone concentrations |
| Text S10 | Model validation |
| Figure S10 | Model validation for ozone concentrations |
| Text S11 | Time profiles of ozone concentration changes in Yokohama |
| Figure S11 | Ozone concentration changes from base scenario to four scenarios in Yokohama |
| Text S12 | Time profiles of ozone concentration changes in Chiba |
| Figure S12 | Ozone concentration changes from base scenario to four scenarios in Chiba |
| Text S13 | Time profiles of ozone concentration change in Saitama |
| Figure S13 | Ozone concentration changes from base scenario to four scenarios in Saitama |
| Text S14 | Time profiles of ozone concentration change in Utsunomiya |
| Figure S14 | Ozone concentration changes from base scenario to four scenarios in Utsunomiya |
| Text S15 | Time profiles of ozone concentration change in Mito |
| Figure S15 | Ozone concentration changes from base scenario to four scenarios in Mito |
| Text S16 | NOx emission inventories used in this study |
| Figure S16 | The maps of NOx emission inventories for (a) industry (b) vehicles, and (c)all emission sources |
| Text S17 | Annual trend of pollutants in Japan |
| Figure S17 | Annual trends in concentrations in Japan |

**Text S1　*Measurement of exhaust emissions using chassis dynamometer***

Tailpipe emissions of the gasoline and gasoline-hybrid passenger vehicles were measured using a chassis dynamometer (MEIDACS-DY6000, Meidensha Corporation). The driving patterns we conducted in this study for passenger vehicles were JC08 (Figure S1-1), which is a previous Japanese-type approval test mode used until 2017, JC08 with cold start, and Tokyo metropolitan driving pattern No.1–12 (Figures S1-2 and S1-3), which are the modes imitating the driving patterns in the real roads in Tokyo. The total amount of NO_x_, CO, and CO_2_ in the emissions of each vehicle was measured using an automotive emissions analyzer (HORIBA): NO_x_ was measured using the chemical luminescence method and CO and CO_2_ were measured using non-dispersive infrared spectroscopy. Fuel consumption by each vehicle was calculated using the carbon balance method based on the CO_2_, CO, and THC measurements. All the measurements were conducted at the environmental temperature of 25±5°C and humidity of 30–75%.

The Tokyo Metropolitan Research Institute for Environmental Protection has observed tailpipe emissions from various vehicles including gasoline passenger vehicles, gasoline-hybrid passenger vehicles, diesel passenger vehicles, diesel heavy-duty vehicles, and diesel buses since 1976.


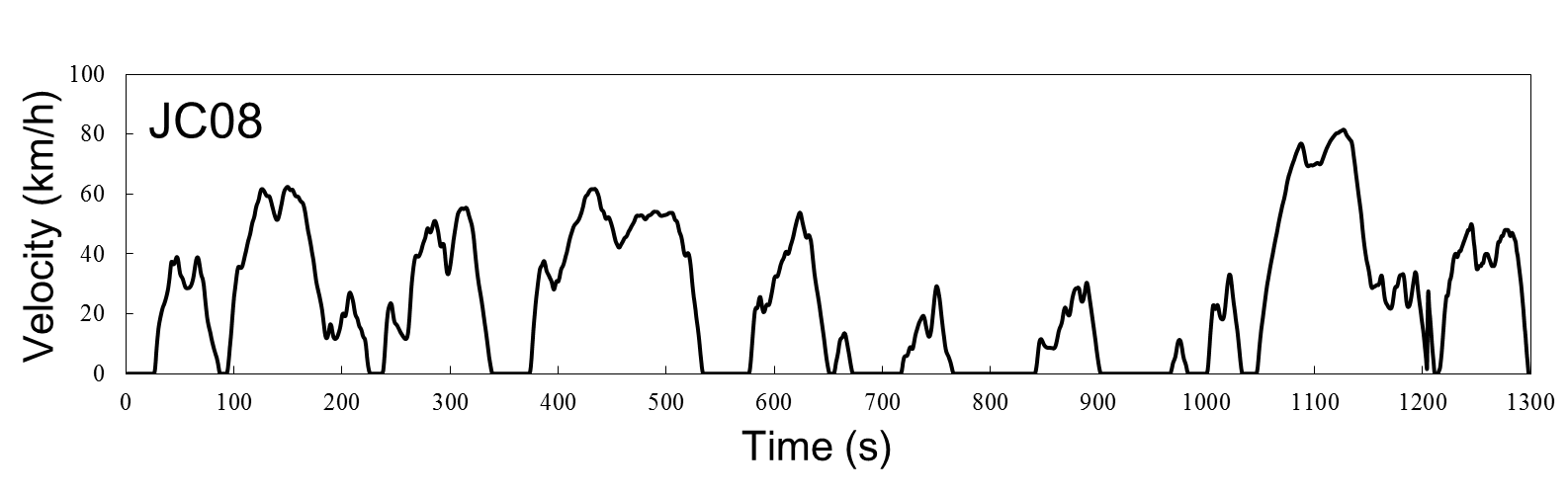


Figure S1-1: Time profile of JC08.


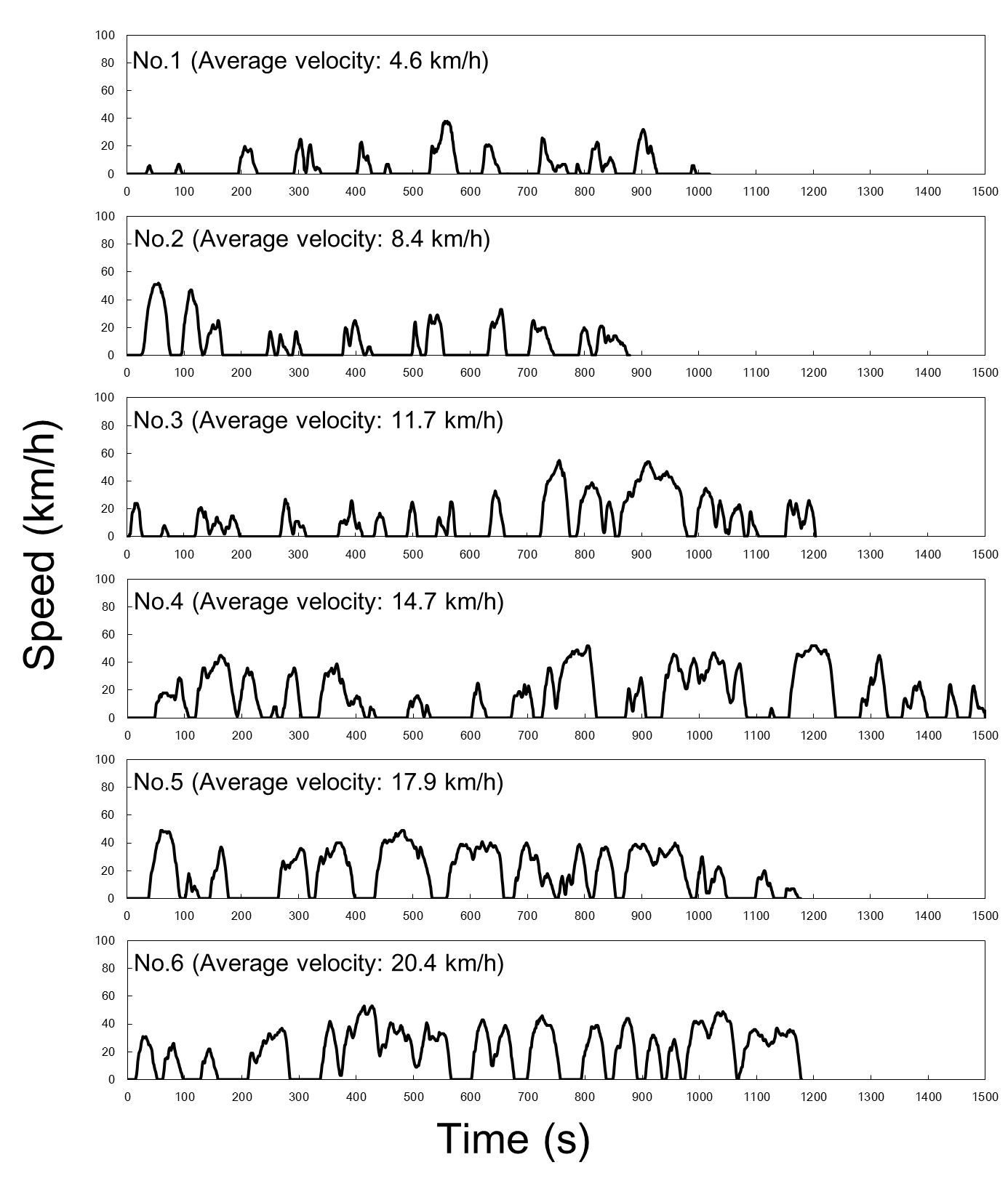


Figure S1-2: Time profiles of Tokyo metropolitan area driving patterns No.1–6.


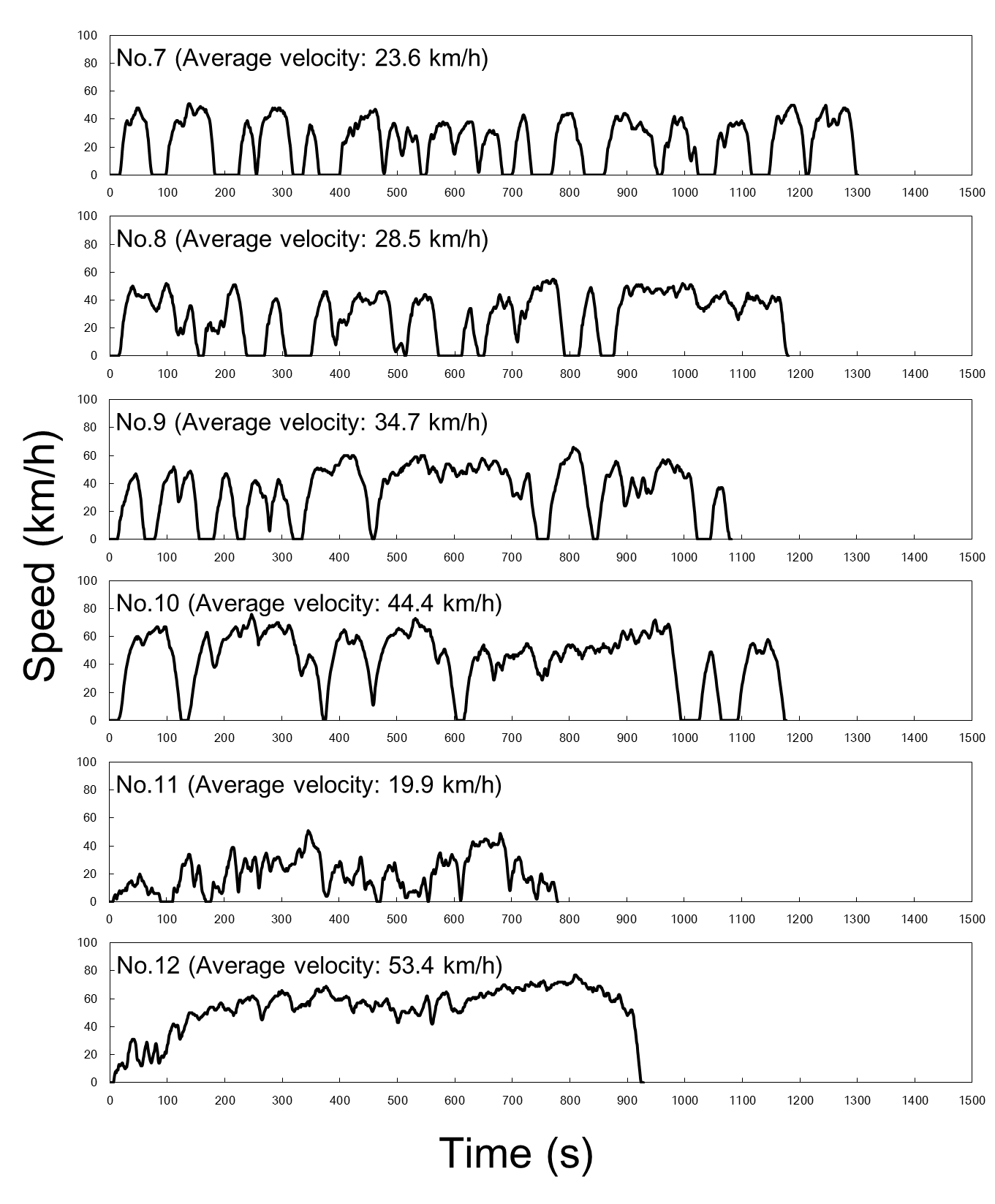


Figure S1-3: Time profiles of Tokyo metropolitan area driving patterns No.7–12.

**Text S2　*Composition analysis of VOCs from tailpipe exhaust emissions***

Total NMHC was determined by the summing the concentrations of 76 VOCs, which were analyzed by Gas Chromatography-Mass Spectrometry and Flame Ionization Detector (GC-MS/FID, 7890 GC system with 5975C inert MSD or Shimadzu GC-2020Plus) for non-oxidized VOCs and Liquid Chromatography-Mass Spectrometry (LC-MS, Agilent1260) for aldehydes and ketone. The analysis period was 2014–2017 for 36 passenger vehicles and 28 heavy-duty vehicles. The detailed information on the analysis, such as the time distribution of the column temperature of GC-MS/FID and LC-MS, is described in Tables S2-1, S2-2, and S2-3.

Table S2-1: Experimental setup for GC-FID.

Table S2-2: Experimental setup for GC-MS

Table S2-3: Experimental setup for LC-MS

**Text S3　*Tailpipe emissions from gasoline and hybrid passenger vehicles***

The experimental results for hot-start emissions were plotted as a function of the average velocity of the vehicle, determined by regression analysis. The empirical function of vehicle emissions is described in equation (S1).

$$C^{i}\left( v \right)=a_{0}^{i}+a_{1}^{i}v+a_{2}^{i}v^{2}+\frac{a_{3}^{i}}{v} (S1)$$

where *C* and *a_j_* (*j*=0–3) represent the emissions and regression coefficients, respectively. The suffix *i* shows the pollutant type (NO_x_, CO, and HC) and fuel consumption. Figure S3-1 shows the relationship between hot-start tailpipe emissions and fuel consumption by gasoline and hybrid passenger vehicles in the Japanese market. The cold-start emissions were determined by the measurement of the JC08 cold-start mode for each vehicle and the average emissions from both gasoline and hybrid vehicles are shown in Figure S3-2.

All the data from gasoline passenger vehicles used in Figures S3-1 and S3-2 were the mean averaged regulation age of vehicles from 1976 to 2015. The distribution of vehicles’ average age in recent years in Japan was cited from a report of the Tokyo metropolitan government^1^. Emissions from gasoline vehicles for each regulation age were determined by the chassis dynamometer measurements conducted by our research institute since 1976.

**Text S3 Reference**

1. Modern Planning Inc. The report of the evaluation of vehicle exhaust emissions in Tokyo metropolitan area in 2016. Published for the Tokyo metropolitan government (2017) (in Japanese).


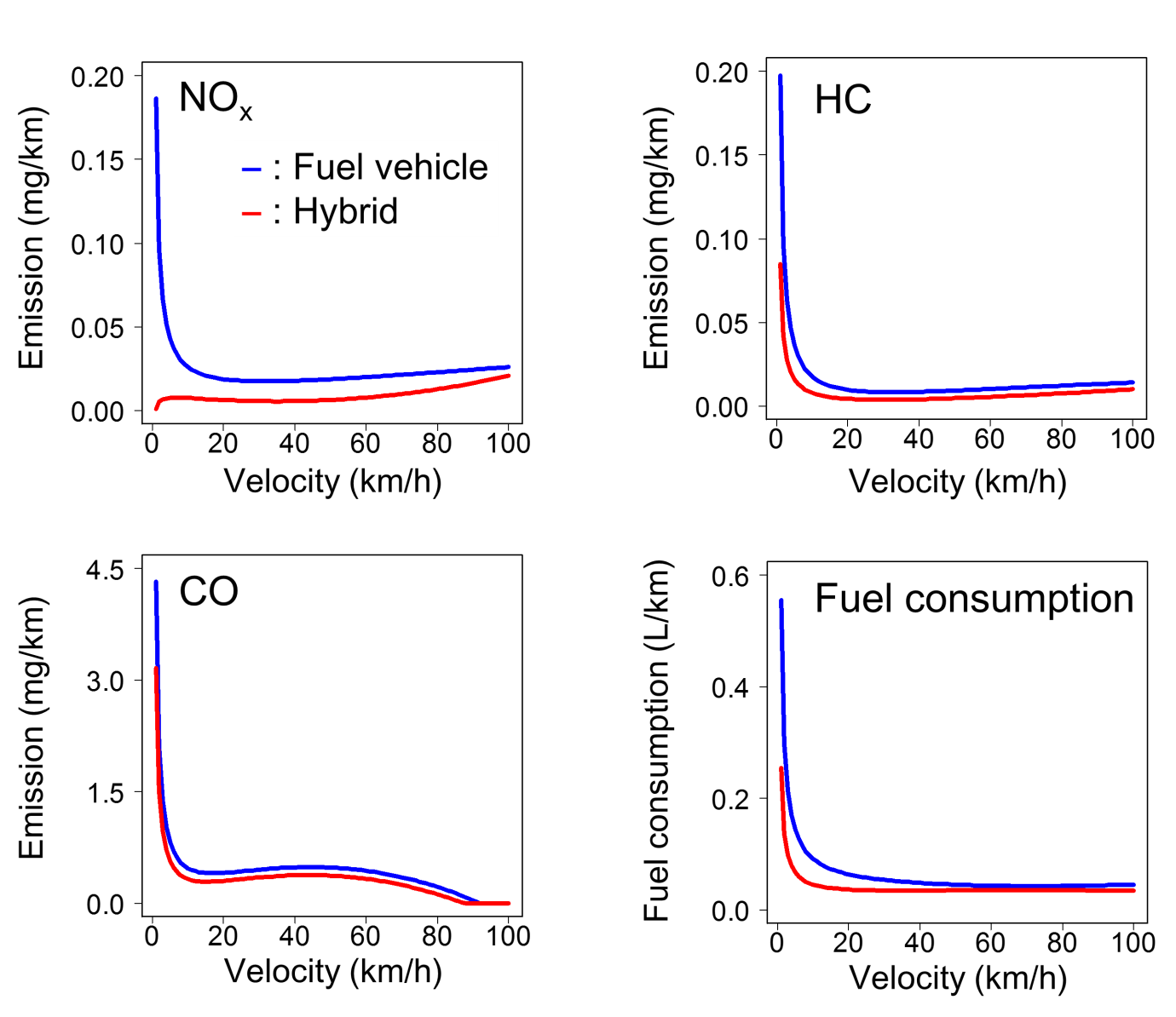


Figure S3-1: Relationships between hot-start tailpipe emissions (or fuel consumption) and vehicle velocity.


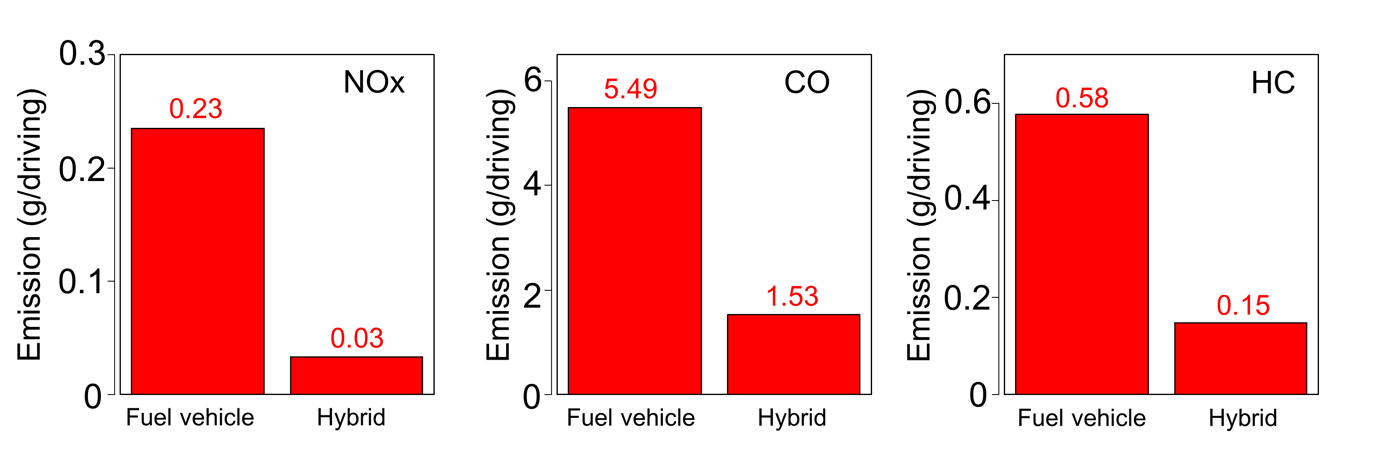


Figure S3-2: Comparison of cold-start emissions of JC08 between gasoline and hybrid vehicles.

**Text S4　*Determination of tailpipe emission factors for gasoline and hybrid passenger vehicles***

Figure S4 shows the distribution of vehicle velocity in Tokyo, estimated from the statistical data published in a report of the Ministry of Land, Infrastructure, Transport, and Tourism in Japan. Using the distribution in Figure S4 and equation (S1), hot-start emissions and average fuel consumption for gasoline and hybrid vehicles were calculated using equation (S2).

$$E^{i}=\int C^{i}\left( v \right)P\left( v \right)dv (S2)$$

where *E^i^* and *P* are the velocity averaged hot-start emissions for *i* (*i*= NO_x_, CO, HC and fuel consumption), respectively, and the vehicle velocity distribution function formulated by the value showed in Figure S4. Finally, the ratio of *E^i^* between gasoline and hybrid vehicles was calculated and used as the emission factor of hybrid vehicle introduction scenarios, SH and AH. Cold-start emission factors for hybrid vehicle introduction scenarios were estimated using the ratio of the cold start emissions between gasoline and hybrid vehicles (Figure S3-2). The method of calculating the emission factors for evaporative emissions is described in the manuscript. All the emission factors used in this study are listed in Tables S4-1 and S4-2.

Table S4-1: Emission factors of the hybrid vehicle introduction scenario. DBL, HSL and RL represent the diurnal breathing loss and hot soak loss and running loss respectively.

Table S4-2: Emission factors of zero-emission vehicle introduction scenarios. DBL, HSL and RL represent the diurnal breathing loss and hot soak loss and running loss respectively.

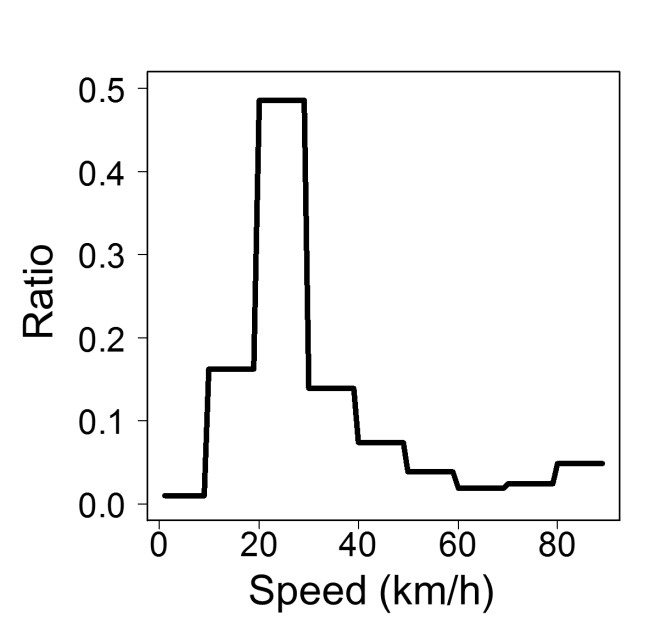


Figure S4: The average distribution of vehicle velocity in Tokyo area.

**Text S5　*VOC composition from tailpipe emission***

The results of the composition analysis of the tailpipe emissions from passenger vehicles and heavy-duty vehicles using GC-MS/FID and LC-MS listed in Tables S2-1, S2-2, and S2-3 are shown in Tables S5-1 and S5-2.

Table S5-1: The average VOC composition of tailpipe emissions from gasoline and hybrid passenger vehicles (mg/km).

Table S5-2: The average VOC composition of tailpipe emissions from diesel heavy-duty vehicles (mg/km).

**Text S6　*VOC composition of evaporative emissions***

The VOC composition used in the SAPRC07 calculation was obtained from our previous study^1^ and the composition ratio is listed in Table S6.

Table S6: The average VOC composition ratio of evaporative emissions of HSL and DBL (g%).

**Text S6 Reference**

1. Hata, H. *et al*. Estimation model for evaporative emissions from gasoline vehicles based on thermodynamics. *Sci. Total Environ*. **618**, 1685-1691, <https://doi.org/10.1016/j.scitotenv.2017.10.030> (2018).

**Text S7　*Distribution of VOC composition analysis results in SAPRC07 form***

Using the VOC composition of the tailpipe and evaporative emissions, the composition ratio using the SAPRC07 form^1^ was calculated.

Table S7-1: VOC distribution of tailpipe emissions from passenger vehicles in SAPRC07 form.

Table S7-2: VOC distribution of tailpipe emissions from heavy-duty vehicles in SAPRC07 form.

Table S7-3: VOC distribution of evaporative emissions in SAPRC07 form.

**Text S7 Reference**

1. Carter, W. P. L. Development of the SAPRC-07 chemical mechanism. *Atmos. Environ*. **44**, 5324-5335. <https://doi.org/10.1016/j.atmosenv.2010.01.026> (2010).

**Text S8　*Calculation conditions of WRF and CMAQ***

The physical models WRF and CMAQ used in this study were based on the conditions described by the Japan’s Study for Reference Air Quality Modeling (J-STREAM)^1^ group and details are presented in Tables S8-1 and S8-2.

Table S8-1: The calculation conditions of WRF.

Table S8-2: The calculation conditions of CMAQ.

**Text S8 Reference**

1. Chatani, S. *et al.* Overview of Model Inter-Comparison in Japan’s Study for Reference Air Quality Modeling (J-STREAM). *Atmosphere*. **9**, 19. <https://doi.org/10.3390/atmos9010019> (2018).

**Text S9　*Calculation results of base scenario***

The ozone concentrations calculated for the base scenario (BASE) are displayed in Figure S9 (average value of the analyzed term from July 1 to August 31, 2013).


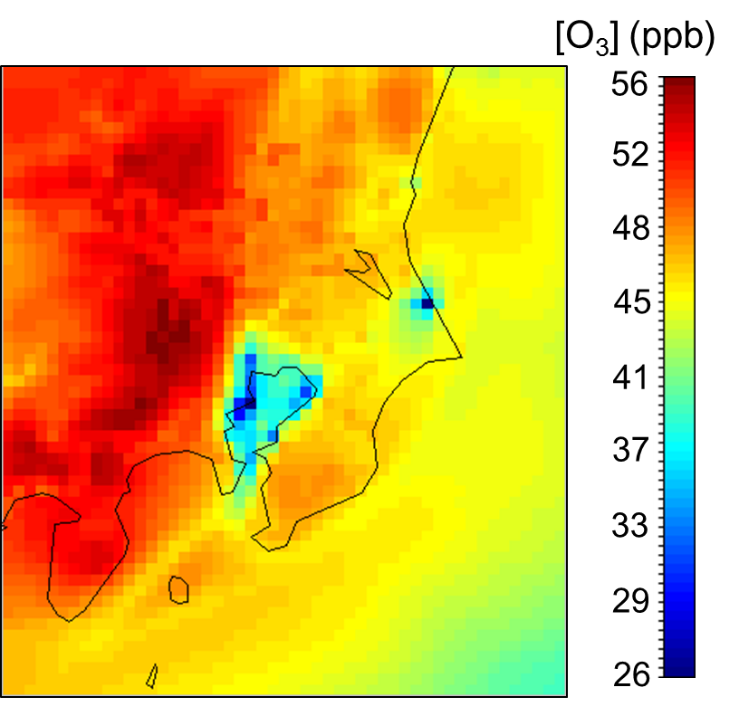


Figure S9: Calculation results of base scenario for average ozone concentrations.

**Text S10　*Model validation***

Figure S10 shows the comparison between the photochemical ozone concentrations in the observation data and the calculation results for BASE for the capital cities of seven prefectures in the Kanto region, Shinjuku (Tokyo), Yokohama (Kanagawa), Chiba (Chiba), Saitama (Saitama), Utsunomiya (Tochigi), Maebashi (Gunma), and Mito (Ibaraki). The analysis period is July 1 to August 31, 2013. The parameters of R and RMSE described in Figure S10 are the correlation coefficient and root mean square error, respectively. The calculated values agree well with the observed data in all areas except for Maebashi. The errors in ozone concentration in Maebashi mainly occurred at nighttime when the ozone is consumed because of the NO_x_ titration effect. According to a previous study conducted by Morino et al.^1^, the chemical reaction rate of NO_x_ titration is high and this leads to high heterogeneity of the calculated grid. This means that the calculated grid size around 4 to 6 km sometimes may not be able to resolute the emission sources enough in the observed area. Despite the discussion of the error in Maebashi, the correlation coefficient between observed and calculated results is above 0.5: this indicates good agreement in the overall trend for the ozone concentration. Thus, we concluded that the calculated model is efficient for the sensitive analysis of this study.

**Text S10 Reference**

1. Morino, Y. *et al.* Inter-comparison of Chemical Transport Models and Evaluation of Model Performance for O_3_ and PM_2.5_ Prediction – Case Study in the Kanto Area in Summer 2007. *J. Jpn. Soc. Atmos. Environ*. **45**, No.5 (2010) (in Japanese with English abstract).


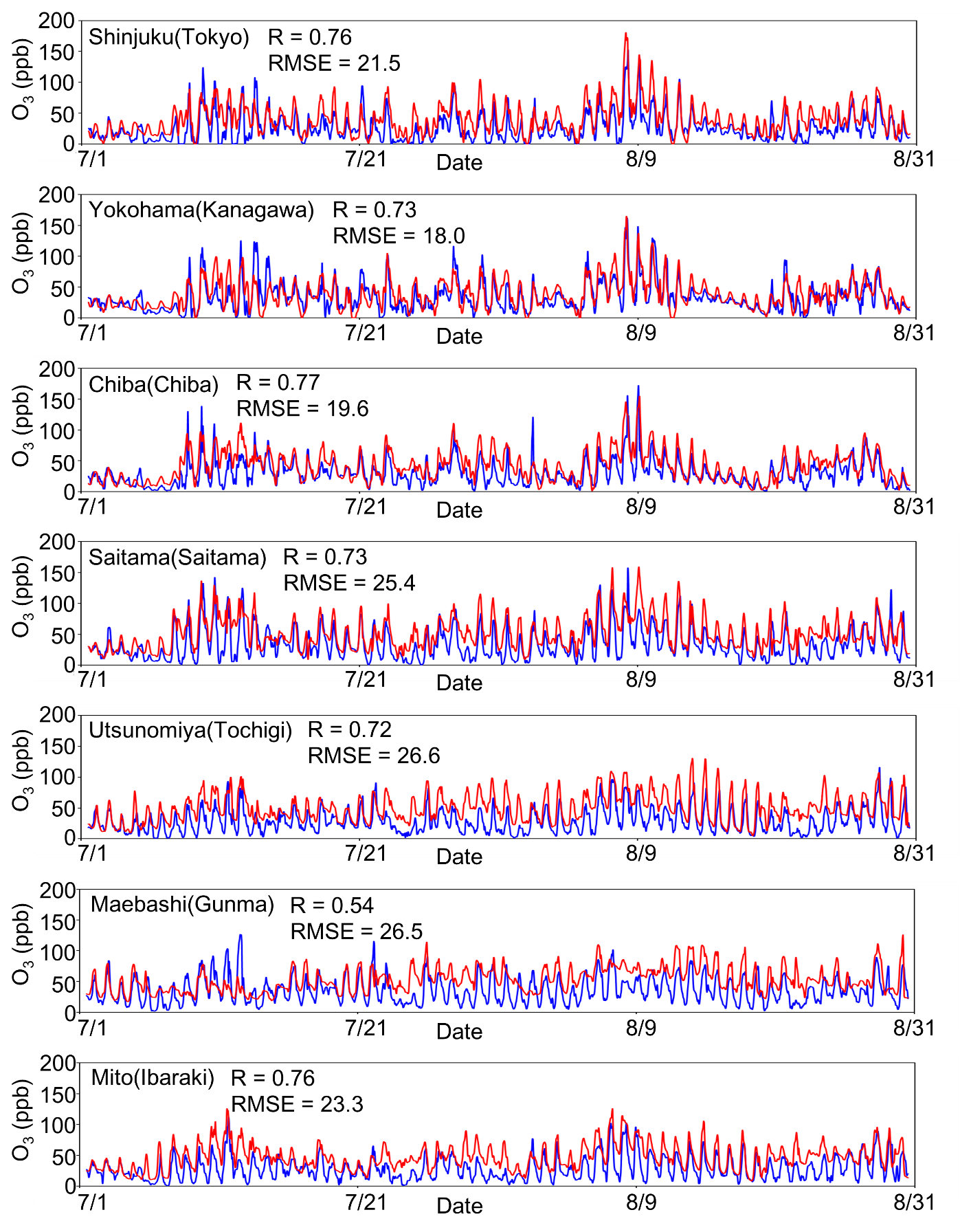


Figure S10: Model validation for ozone concentrations (**■**: Observed, **■**: Modeled).

**Text S11　*Time profiles of ozone concentration changes in Yokohama***

Figure S11 shows the ozone concentration changes in Yokohama when the next-generation vehicles are introduced.


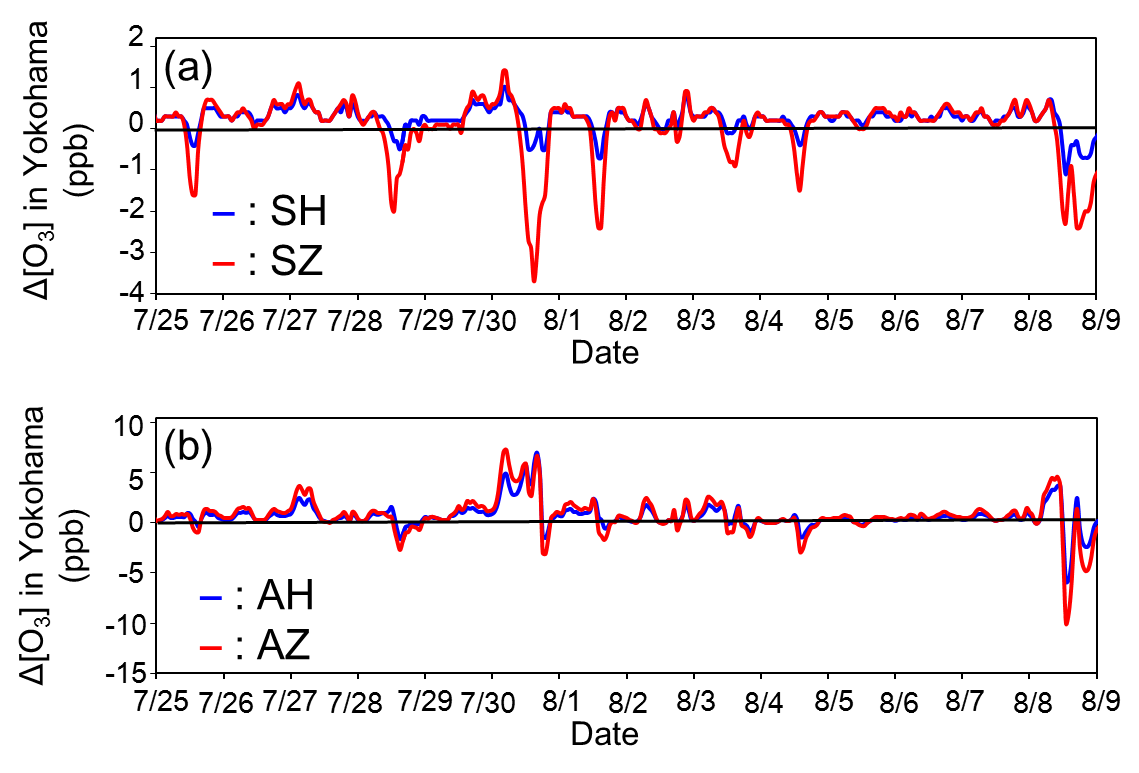


Figure S11: Ozone concentration changes from base scenario to four scenarios in Yokohama (a) SH and SZ, (b) AH and AZ.

**Text S12　*Time profiles of ozone concentration changes in Chiba***

Figure S12 shows the ozone concentration changes in Chiba when the next-generation vehicles are introduced.


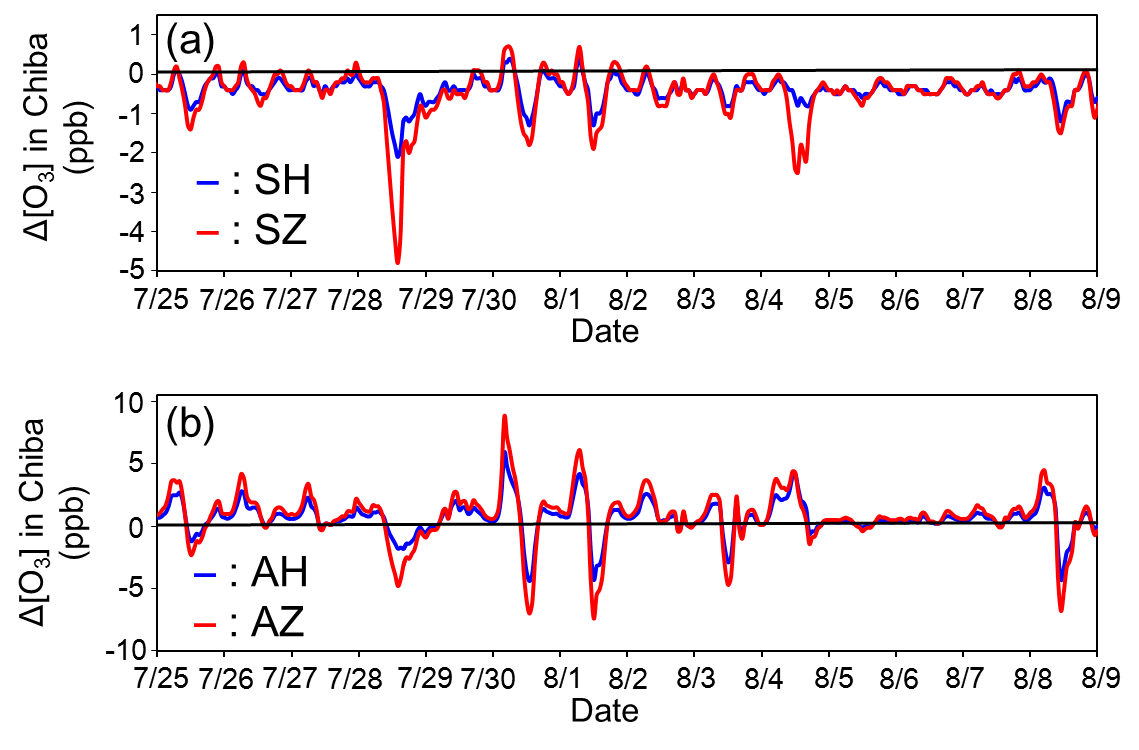


Figure S12: Ozone concentration changes from base scenario to four scenarios in Chiba (a) SH and SZ, (b) AH and AZ.

**Text S13　*Time profiles of ozone concentration change in Saitama***

Figure S13 shows the ozone concentration changes in Saitama when the next-generation vehicles are introduced.


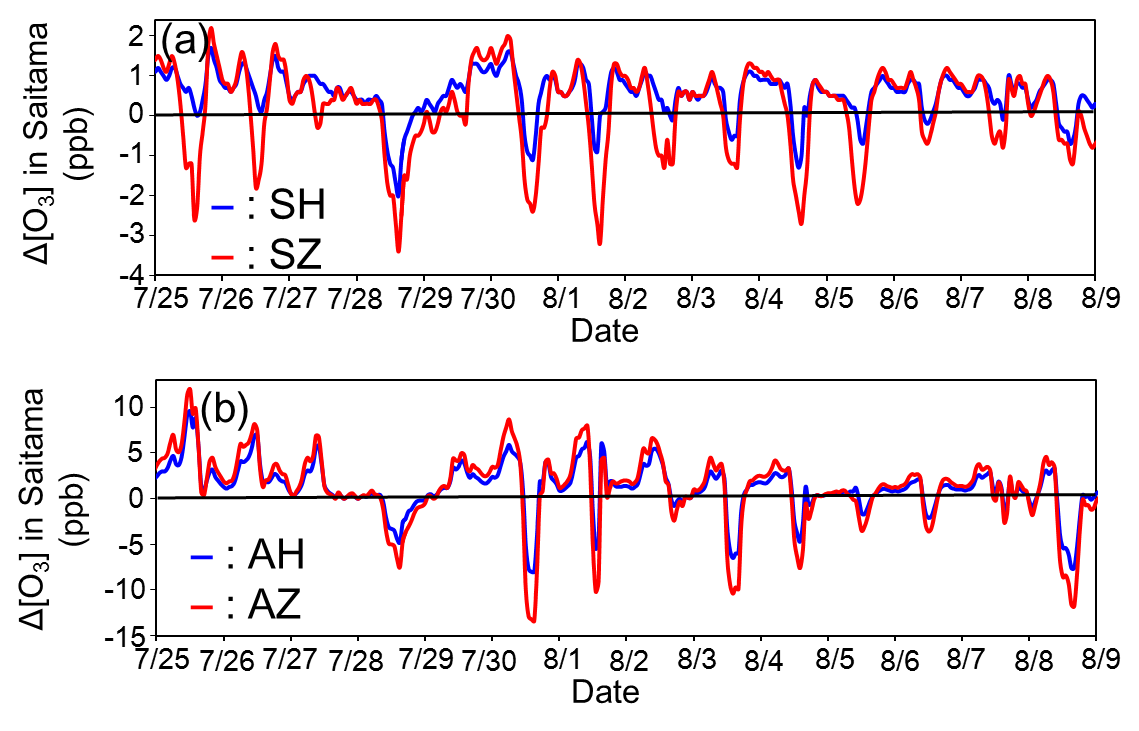


Figure S13: Ozone concentration changes from base scenario to four scenarios in Saitama (a) SH and SZ, (b) AH and AZ.

**Text S14　*Time profiles of ozone concentration change in Utsunomiya***

Figure S14 shows the ozone concentration changes in Utsunomiya when the next-generation vehicles are introduced.


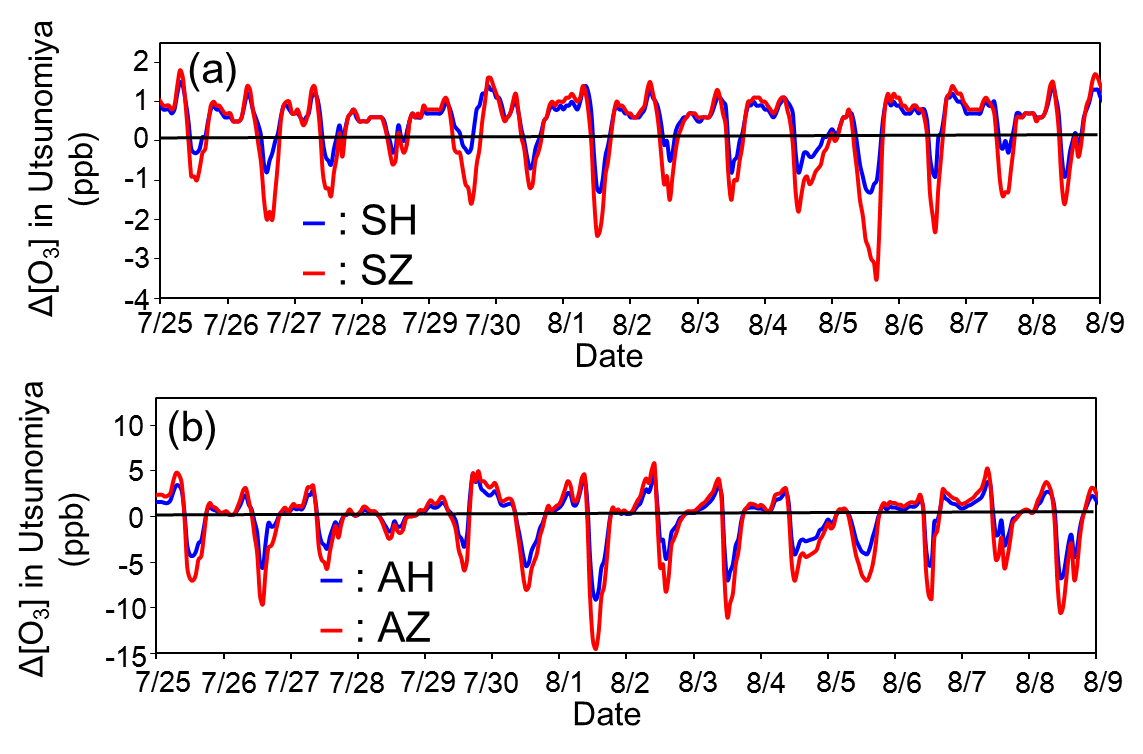


Figure S14: Ozone concentration changes from base scenario to four scenarios in Utsunomiya (a) SH and SZ, (b) AH and AZ.

**Text S15　*Time profiles of ozone concentration change in Mito***

Figure S15 shows the ozone concentration changes in Mito when the next-generation vehicles are introduced.


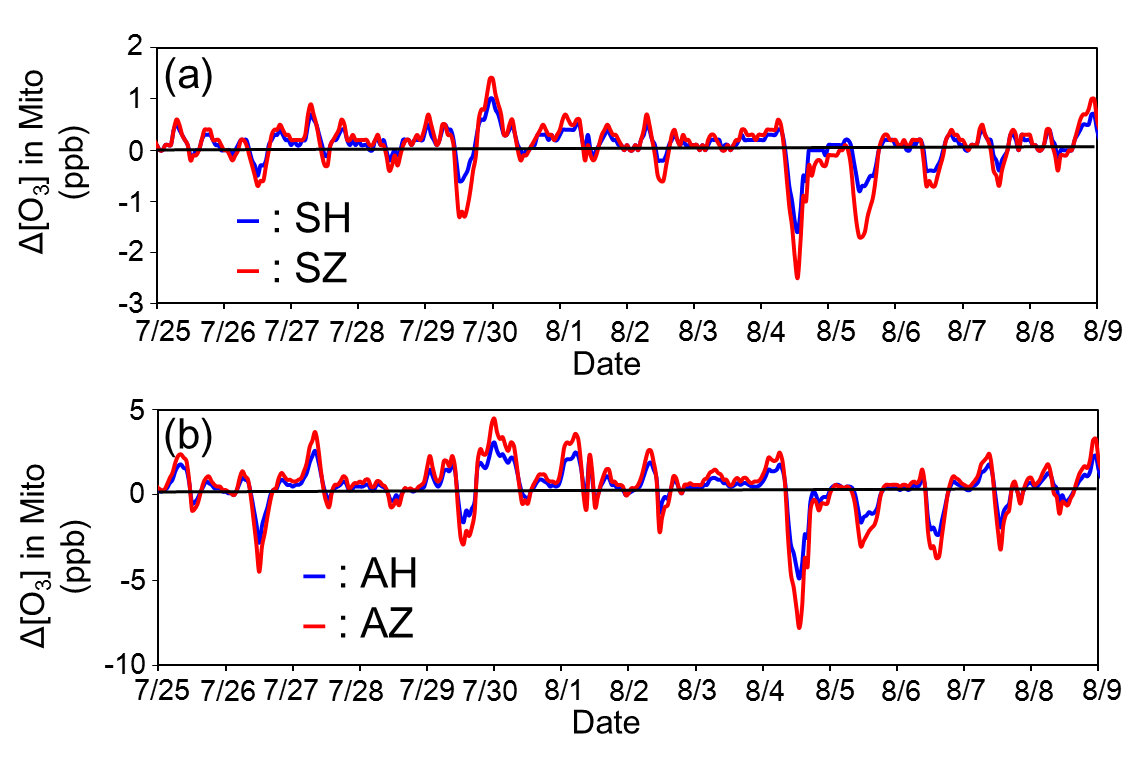


Figure S15: Ozone concentration changes from base scenario to four scenarios in Mito (a) SH and SZ, (b) AH and AZ.

**Text S16　*NO_x_ emission inventories used in this study***

Figure S16 shows the NO_x_ emission inventories from the industry, vehicles, and all emission sources. The area inside the red circle corresponds to Mito, which has relatively high NO_x_ emissions, although it is a suburban area.


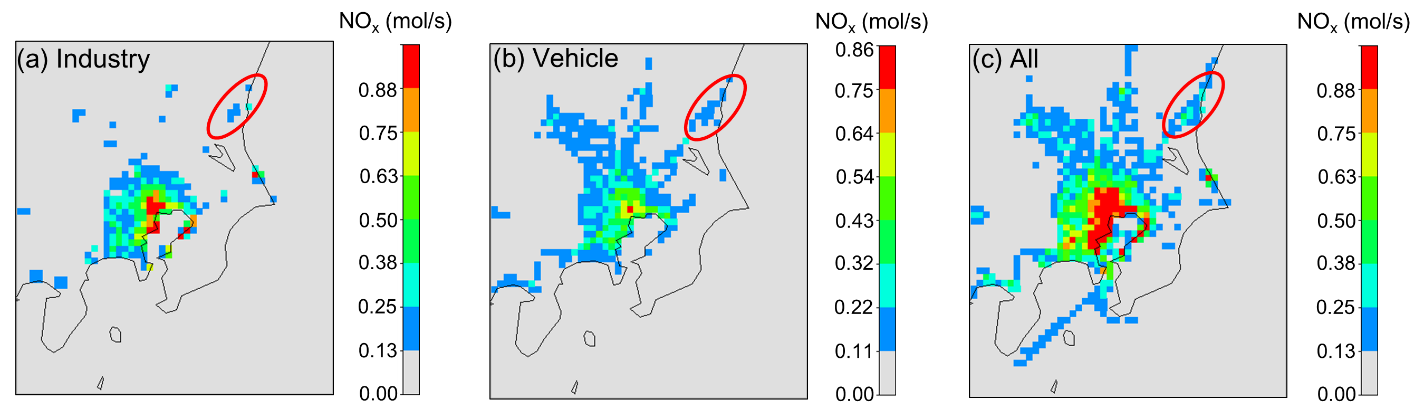


Figure S16: The maps of NO_x_ emission inventories for (a) industry (b) vehicles, and (c) all emission sources.

**Text S17　*Annual trend of pollutants in Japan***

Figure S17 shows the trends of the annual average concentrations of NO_x_, VOCs, and ozone^1^. Although NO_x_ and VOC concentrations decrease in time, ozone concentrations are gradually increasing.


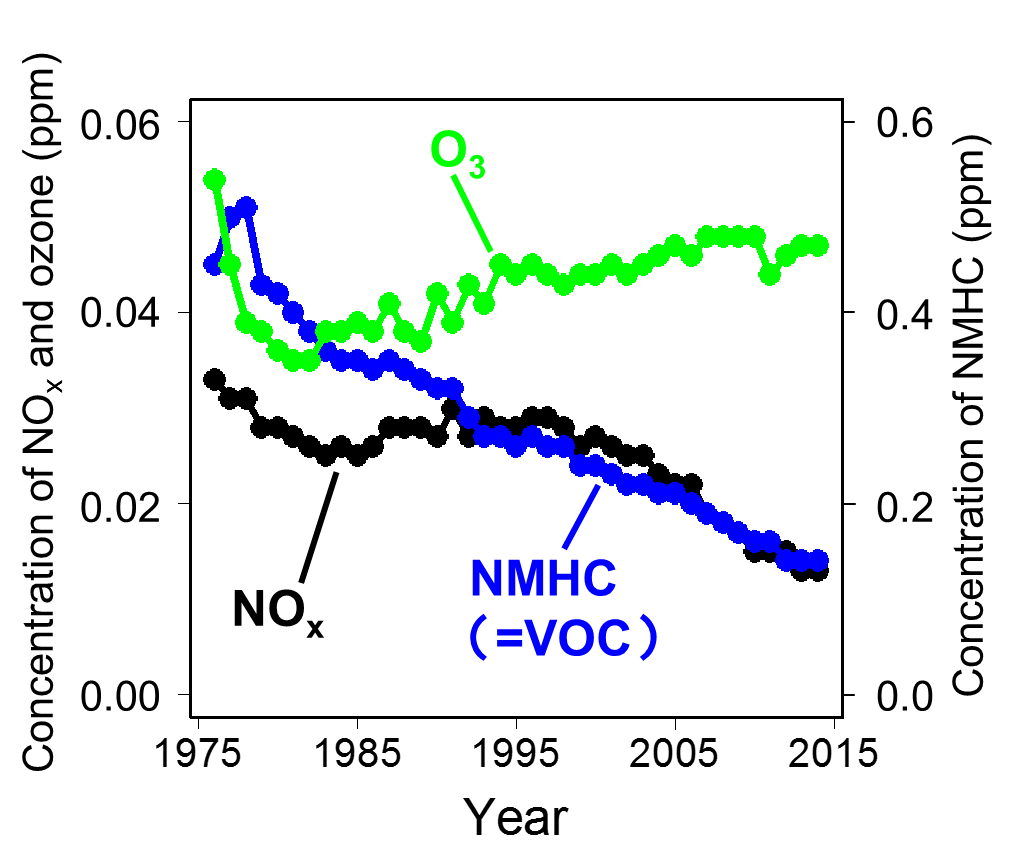


Figure S17: Annual trends in concentrations in Japan.

**Text S17 Reference**

1. National Institute for Environmental Studies. https//www.nies.go.jp/igreen.
